# Antibiotic resistance shaping multi-level population biology of bacteria

**DOI:** 10.3389/fmicb.2013.00015

**Published:** 2013-03-06

**Authors:** Fernando Baquero, Ana P. Tedim, Teresa M. Coque

**Affiliations:** ^1^Department of Microbiology, Hospital Universitario Ramón y Cajal, Instituto Ramón y Cajal de Investigación SanitariaMadrid, Spain; ^2^Centros de Investigación Biomédica en Red de Epidemiología y Salud PúblicaMadrid, Spain; ^3^Unidad de Resistencia a Antibióticos y Virulencia Bacteriana asociada al Consejo Superior de Investigaciones CientíficasMadrid, Spain

**Keywords:** antibiotics, resistance, population biology, multi-level selection, evolution, evolvability, resistome, microbiome

## Abstract

Antibiotics have natural functions, mostly involving cell-to-cell signaling networks. The anthropogenic production of antibiotics, and its release in the microbiosphere results in a disturbance of these networks, antibiotic resistance tending to preserve its integrity. The cost of such adaptation is the emergence and dissemination of antibiotic resistance genes, and of all genetic and cellular vehicles in which these genes are located. Selection of the combinations of the different evolutionary units (genes, integrons, transposons, plasmids, cells, communities and microbiomes, hosts) is highly asymmetrical. Each unit of selection is a self-interested entity, exploiting the higher hierarchical unit for its own benefit, but in doing so the higher hierarchical unit might acquire critical traits for its spread because of the exploitation of the lower hierarchical unit. This interactive trade-off shapes the population biology of antibiotic resistance, a composed-complex array of the independent “population biologies.” Antibiotics modify the abundance and the interactive field of each of these units. Antibiotics increase the number and evolvability of “clinical” antibiotic resistance genes, but probably also many other genes with different primary functions but with a resistance phenotype present in the environmental resistome. Antibiotics influence the abundance, modularity, and spread of integrons, transposons, and plasmids, mostly acting on structures present before the antibiotic era. Antibiotics enrich particular bacterial lineages and clones and contribute to local clonalization processes. Antibiotics amplify particular genetic exchange communities sharing antibiotic resistance genes and platforms within microbiomes. In particular human or animal hosts, the microbiomic composition might facilitate the interactions between evolutionary units involved in antibiotic resistance. The understanding of antibiotic resistance implies expanding our knowledge on multi-level population biology of bacteria.

Antibiotics produced by natural organisms play a role in their interactions shaping the lifestyle and homeostasis of bacterial populations and communities (Waksman, 1961; Davies, 2006; Fajardo and Martínez, 2008; Aminov, 2009). Such interactions might be of antagonistic nature as the production of antibiotics serves to inhibit other bacterial populations. Inhibition does not necessarily intend to kill competitive bacterial organisms, but rather prevent undesirable local overgrowth of partners in shared ecosystems. The diffusion of antibiotics in the environment assures an “exclusive zone” at a certain distance from the producer population. At the borders of such a zone, the potentially competing organisms are confronted with very low antibiotic concentrations, probably sufficient to decrease their growth rate, but not to kill the competing neighbor. In this sense, it is highly possible that the production of antagonistic (allelopathic) substances by microorganisms has more a defensive than offensive nature (Chao and Levin, 1981). In addition, mutual inhibition is frequently desirable for the maintenance of healthy species diversity in a particular ecosystem (Czárán et al., 2002; Becker et al., 2012; Cordero et al., 2012). It is of note that natural antibiotic production, decreasing the growth rate of the competing population, not only restricts its local predominance, but also assures that this population is preserved, as antibiotic-promoted cessation of growth is a highly effective system to avoid antibiotic killing.

However, the production of natural antibiotics might only have functions unrelated with inter-bacterial antagonisms. Antagonism might arise in particular contexts as a side-effect of cell-to-cell signaling effects resulting in self-regulation of growth, virulence, sporulation, motility, mutagenesis, SOS stress response, phage induction, transformation, lateral gene transfer, intra-chromosomal recombination, or biofilm formation (Goh et al., 2002; Ubeda et al., 2005; Linares et al., 2006; Yim et al., 2007; Martínez, 2008; Couce and Blázquez, 2009; Kohanski et al., 2010; Allen et al., 2011; Baharoglu and Mazel, 2011; Pedró et al., 2011; Looft and Allen, 2012; Looft et al., 2012).

Natural antibiotic resistance modulates the effect of the natural production of antibiotics, so antibiotic production and antibiotic resistance act as two complementary sides of the same process assuring the homeostasis of microbial populations and communities. In fact, communities with a cohesive habitat association might act as units in terms of antibiotic production and resistance. In these clusters, antibiotics are frequently produced by few bacterial organisms, whereas other members of the club are resistant (Cordero et al., 2012).

As in the case of natural antibiotic production, natural antibiotic resistance might not only focus on “defense against antibiotics” or “self-protection” in antibiotic producers. In fact, this “defense” is frequently a side-effect of other functions of the “natural resistance mechanisms,” including nutrition, metabolism, detoxification of noxious substances, and catabolic processes (Dantas et al., 2008; Martínez, 2008; Alvarez-Ortega et al., 2011; Martínez and Rojo, 2011; Qu and Spain, 2011).

The so-called “intrinsic resistome,” the ensemble of non-acquired genes and functions normally present in bacterial cells which influence the susceptibility to antibiotics, might account for 3% of the bacterial genome (Fajardo et al., 2008). Obviously, such a huge number of “defensive” genes reflects the unspecific nature of their functions in which antibiotic resistance is concerned (Fajardo et al., 2008; Alvarez-Ortega et al., 2011). That does not mean that these genes were not submitted to horizontal gene transfer before the crisis provoked by the industrial antibiotics pollution, illustrating that besides direct selection by clinical antibiotics other factors contribute to dissemination and maintenance of antibiotic resistance genes in bacterial populations (Biel and Hartl, 1983; Aminov and Mackie, 2007; Mindlin et al., 2008; Allen et al., 2009; D'Costa et al., 2011). In any case, it has been recently suggested that the close identity of resistance genes (and resistance platforms) from clinical strains and environmental strains might indicate recent exchange events (Forsberg et al., 2012). From this perspective, natural antibiotics and antibiotic “resistance” mechanisms have a natural regulatory role in shaping both population biology and evolutionary biology of bacterial organisms. However, the amount of active antibiotic determining this physiological natural “antibiotic environment” is extremely low (Halling-Sørensen et al., 1998). Of course antibiotic-producer organisms are endowed with “mechanisms for self-protection,” which have been considered as the origin of modern functions involved in clinical antibiotic resistance (Benveniste and Davies, 1973). However, phylogenetic studies suggest that current clinical resistance genes are not found in antibiotic producers, and its emergence in clinical strains cannot be explained by recent horizontal gene transfer from these organisms. Nevertheless, they might have been historically submitted to duplications and frequent horizontal gene transfer, so that “modern” resistance genes might have evolved along complex evolutionary processes pre-existing the industrial release of antibiotics. In fact many identical “resistance genes” are found in environmental and clinical organisms (Cantón et al., 1999; Forsberg et al., 2012). In any case, what we denominate “antibiotic resistance” for clinical microorganisms is extremely rare in nature; other kinds of “resistance” genes, those of the “intrinsic resistome,” able to protect cells from tiny concentrations of natural antibiotics, dominate in the wild environments.

The main problem that we are examining in this manuscript derives from the huge escalation of the amount of antibiotics released into the microbial environments as an effect of anthropogenic action, greatly exceeding the amount of natural antibiotics signaling and controlling the homeostasis of the bacterial world. Moreover, the amount of antimicrobials of anthropogenic origin entering into the environment assures the presence of every possible antibiotic concentration in contact with bacteria. The consequences of such an extensive release of inhibitory and regulatory molecules have an important impact on the population biology and evolutionary biology of bacteria.

## POPULATION BIOLOGY OF THE UNITS OF SELECTION

The units of selection define the evolutionary individuals (Lewontin, 1970; Brandon, 1987; Mayr, 1997; Okasha, 2004; Dupré and O'Malley, 2007; Lloyd, 2008; Doolittle and Zhaxybayeva, 2010; Baquero, 2011; Rodriguez-Valera and Ussery, 2012). But what is selected in the case of antibiotic resistance? Possible units of selection in antibiotic resistance are discrete genetic sequences, genes, operons, functional genetic modules, mobile genetic elements (MGEs) as integrons, transposons, integrative–conjugative elements (ICEs), plasmids, or at the cellular and supra-cellular levels, genomes and cells (organisms), clones, clonal complexes, species, communities, and ecosystems. Note that all these possible units belong to different hierarchical levels, ranging from the relatively simple to the complex, as resistance genes are part of integrons, integrons part of transposons, transposons part of plasmids, plasmids part of cells, cells part of clones, clones part of species, and so on. Each unit is a “vessel” for the other(s), affecting not only its potential dissemination but also its rate of introgressive descent and evolution (Doolittle and Zhaxybayeva, 2010; Baquero, 2011; Bapteste et al., 2012; Cordero et al., 2012). The investigation of such a trans-hierarchical landscape clearly requires a multi-level population genetic approach (Baquero and Coque, 2011; Day et al., 2011; Cordero et al., 2012).

What we propose in this work is essentially a mental heuristic exercise. Let us imagine that we are aware of a kind of replicators called genes but we still ignore the existence of cells. We could observe changes in the frequency and variety of genes, and we might consider populations of genes, submitted to evolutionary dynamics and natural selection. If we were only conscious of the existence of transposons, we would establish population biology of transposons. If we considered plasmids, we would refer to the pan-plasmidome, the plasmid population harbored by a particular microbiome or a particular bacterial group (Fondi and Fani, 2010; Mizrahi, 2012). This would apply for every unit of selection. We would of course be able to observe changes in the abundance and variety of each unit involving a resistance trait as a consequence of the presence of antibiotics in the environment. Each unit of selection is a self-interested entity (Rankin et al., 2011b) exploiting the higher hierarchical unit for its own benefit (resistance plasmids exploit successful bacterial clones), but the higher unit might acquire critical traits for its spread because of the exploitation of the lower hierarchy unit (bacterial clones, bacterial communities, or microbiomes might be successful because of resistance plasmids, ICEs, or elements within those; Castillo et al., 2005; Mizrahi, 2012; Novais et al., 2012b). This trade-off of interactions shapes the global population biology of antibiotic resistance. In a certain sense, this multi-level perspective represents a “second line of complexity” in the classic view of antibiotics-driven natural selection processes, from the selection of a resistant cell to multi-level selections. Such processes should increase the absolute density (number) of all pieces involved in antibiotic resistance, and consequently might favor their interactions and emergence of novel combinatorial patterns (Baquero, 2004). Prediction of which pieces and patterns will evolve is a crucial issue for the management of multiantibiotic resistance, and might be possible if we have the right data (Martínez et al., 2007; Baquero, 2011; Partridge, 2011).

## ANTIBIOTICS AND POPULATIONS OF RESISTANCE GENES

Have antibiotics increased the abundance of highly effective resistance genes in the bacterial world? Confronted with antibiotics, bacterial populations might adapt by selecting “more effective” mutants of wild genes endowed with other functions, but providing low level of resistance. Such process is favored by gene duplication, so that wild genes having a “small effect” on resistance could increase in number to increase protection. It is of note that this process might be much more frequent than mutation (Näsvall et al., 2012). A high number of gene-copies might in fact transitorily accumulate during selection, producing a full resistance phenotype. The question is if such gene duplications might contribute to the emergence of novel resistance genes. Once the permanence of a functional copy of a given gene is guaranteed, the second (or *n*-) copy has the evolutionary freedom (liberation from purifying selection) to be modified, eventually leading to a variant or novel gene (Kondrashov et al., 2002; Näsvall et al., 2012). It is of note that not all genes have equal chances of duplication, and certainly there are adaptive genes with a higher potential variability, containing highly variable regions interspersed among well-conserved, “segmentally variable genes,” as ABC transporters involved in multidrug resistance (Zheng et al., 2004). Mutational changes in genes, leading to novel resistance genes, are facilitated under circumstances of enhanced mutagenesis in the host strain. Hyper-mutable bacteria (“mutators”) are enriched in allelic variants of resistance genes, eventually providing wider resistance spectrum, as in the case of beta-lactamases (Baquero et al., 2005). Indeed organisms with enhanced mutation rates (frequently involving failures in the mismatch repair system) see their possibility of survival increased, and inside these strains, other genes could be modified to provide antibiotic resistance. Note that hyper-mutation and gene variation at large, might result from the effects of the antibiotic themselves (Blázquez et al., 2012).

Indeed, intra- and inter-bacterial gene movement and recombinational events between genetic platforms contribute to the total amount of resistance genes. In fact, the “biological success” of a resistance gene is dependent on its wider genetic context (Walsh, 2006; Wozniak and Waldor, 2010; Bertels and Rainey, 2011). Moreover, hybrid resistance genes resulting from recombinational events are not infrequent in nature (Goffin and Ghuysen, 1998; Maiden, 1998; Novais et al., 2012b). Under antibiotic exposure, bacterial pathogens in humans and animals (and commensals) might fix and further refine acquired resistance genes originated in areas less exposed to antimicrobials, as in the soil (including the rhizosphere!), or water bodies (including sewage!) (Aminov, 2009; Lupo et al., 2012).

If antibiotics have polluted the entire globe, including wild environments, specialized antibiotic resistance genes, identical to those found in hospitals, can be found everywhere else, including the most remote and wild regions (Gilliver et al., 1999; Osterblad et al., 2001; Sjölund et al., 2008; Allen et al., 2009; Quinteira et al., 2011; Stalder et al., 2012). However, the variety of natural bacterial genes that can provide antibiotic resistance in a heterologous host is much larger than that actually found in human pathogens (Dantas et al., 2008). Why only a very small fraction of “resistance genes” present in the “global resistome” have entered in human pathogens is a poorly addressed question. Of course genes from phylogenetically remote organisms should have functional connectivity and concert with the host systems, and that certainly constitutes an important bottleneck for their acceptance (Halary et al., 2010; Martínez, 2011). However, relatively “independent” functionally connected gene clusters (Zheng et al., 2005) determining resistance might be better tolerated and eventually fixed (Popa et al., 2011). In any case, as stated recently (Skippington and Ragan, 2011), the network and evolutionary dynamics that allow the stoichiometric participation of horizontally transferred genes in cellular networks remains poorly addressed, even though new bioinformatic advances have recently been made available (Cohen et al., 2012).

Considering potential sets of “acceptable” resistance genes able to evolve in bacterial populations, eventually only the “fittest genes” resulting from competition among genes might finally reach high densities. Competition is expected to occur mostly among orthologs or paralogs (for instance resulting from recent duplications) occupying the same functional niches (Kondrashov et al., 2002; Francino, 2012). Antibiotics could have enriched the more efficient adaptive genes among competing genes (for instance the more detoxifying ones; Novais et al., 2010b). However, the “fittest genes” are not necessarily those with the best intrinsic activity in terms of providing antibiotic resistance. Different resistance genes impose different biological costs for the host strain. As it was stated above, successful novel resistance genes should be fit in a particular genetic context, that is, the epigenetic compatibility of a new gene with the host genome is critical in the acquisition of resistance (Sánchez and Martínez, 2012). Such fitness bottleneck will select, in combination with the detoxifying efficiency, the novel successful genes.

Alternatively, the quantitative success of a particular gene (as *bla*_TEM-1_ in *Escherichia coli*, or *blaZ* in *Staphylococcus aureus*) might result only from a “founder effect” (Livermore, 2000; Martinez et al., 2009), that is, the first gene that by chance conferred a selective advantage in particular conditions was fixed and that resulted in a successful wide spread. This founder effect in human and animal pathogens might have occurred because of multiple selective events in environmental organisms exposed to dynamic landscapes. In fact, founder effects are expected to occur in a continuous and cumulative way (Aguilée et al., 2009). In turn, such emergent events might have been facilitated by changes in environmental conditions (as animal crowding in farms) resulting in local fluctuations in the size of particular bacterial populations, thus favoring acquisition by lateral genetic transfer of adaptive traits from environmental bacteria (Balloux, 2010). Similarly, the changes in the environment, the landscape dynamics, influence the probability of founders fixation, as well as the possibilities for extinction and re-emergence (Aguilée et al., 2011). It is not impossible that many resistance genes, even those rarely found or never found in the clinical environment, could have also been enriched by environmental antibiotic exposure (Martinez et al., 2009; Sommer et al., 2009).

How can we explain that the same resistance gene of plausible environmental origin (as *bla*_CTX-M__s_ or *bla*_CMY__s_) appears to have been captured in separate events by different gene-capturing elements as IS*Ecp*, IS*BR1*, or IS*26*? (Barlow and Hall, 2002; Toleman et al., 2006; Valverde et al., 2009; Partridge, 2011). There is a contemporary enrichment in organisms as Enterobacteriaceae of captured gene(s) with apparently the same function, and a bloom of a diversity of new genes coding for resistance to beta-lactams (Cantón and Coque, 2006; Coque et al., 2008; Poirel et al., 2012). This might be due to a dense interactive field resulting from an in the number of environmental species (donors) where it originated from, as well as an environmental increase in population density of a variety of “good recipients” as *E. coli* or *Klebsiella pneumoniae*. Recipients increase could result from both the augmentation of the total number of Enterobacteriaceae in the gut microbiota of mammals, probably due to antibiotic exposure, and the massive release of human-and animal sewage to the environment (see later).

Other less-successful resistance or pre-resistance genes might not be relevant in the clinical setting, but constitute an increasing reservoir of unpredictable consequences, and undoubtedly might influence the population ecology of bacteria. On the other hand, the selection of variant genes might occur at very low antibiotic concentrations (Henderson-Begg et al., 2006; Gullberg et al., 2011) particularly among natural concentration gradients (Baquero and Negri, 1997; Negri et al., 2000; Hermsen et al., 2012). It can be suggested that the overall increase in the amount of resistance genes on Earth also has a positive effect in maintaining the desirable homeostasis of bacterial populations in a heavily antibiotic-polluted environment.

An interesting point in gene population biology is the question of why a number of resistance genes maintain their full sequence integrity through myriads of replications in spite of an apparently insufficient level of antibiotic selection. Even considering that they are co-selected with genes under active selection (for instance being part of the collection of gene cassettes of an integron), their functionality seems to be better preserved than could be expected. This might suggest that the current function of a number of classic “resistance genes” is something other than antibiotic resistance (exaptation; Alonso and Gready, 2006; Petrova et al., 2011; Sánchez and Martínez, 2012).

Resistance genes tend to be collected in particular clones and clustered in common genetic platforms (Partridge and Hall, 2004; Ktari et al., 2006; Partridge, 2011; Potron et al., 2013), probably following the “genetic capitalism principle,” that is, the more resistant clones and the most fit resistance-providing platforms are selected, and then their ability to acquire novel adaptive traits is favored (Lawrence, 1997; Baquero, 2004). As we will see along this review, the extensive antibiotic-promoted selection of resistant bacterial organisms is a selection of the “vehicles” where antibiotic resistance genes are located, and the selection of “vehicles” assures the selection of the genes that they contain. Consequently, the total amount of resistance genes should increase under selection. Interestingly, some “vehicles” (as MGEs are more frequently associated with resistance genes than others. As complementary explanations, we can recall here the founder effect (advantages for the first gene-capturing MGEs), the influence of genes and platforms on the overall fitness of the recipient cells, or the higher prevalence of particular MGEs in the organisms more exposed to antibiotics, heavy metals, biocides, or other ecological stressors (Stokes and Gillings, 2011).

## ANTIBIOTICS AND POPULATIONS OF RESISTANCE INTEGRONS

Have antibiotics increased the abundance, in the microbial world, of integrons able to capture resistance genes? Integrons possess a site-specific recombination system able to integrate, rearrange, and express adaptive genes, including antibiotic resistance genes (Mazel, 2006; Partridge, 2011; Stokes and Gillings, 2011; Moura et al., 2012). These genetic platforms are ancient structures (several hundred million years old) that were already involved in the initial outbreaks of antibiotic resistance in the 1950s (Liebert et al., 1999; Mazel, 2006; Revilla et al., 2008). The same type of integrons, now carrying a diversity of antibiotic resistance genes, are preserved in the current bacterial world, and have installed themselves in natural environments along extended periods of time (Petrova et al., 2011; Stokes and Gillings, 2011; Stalder et al., 2012). Integrons evolution often results in the local array of resistance genes, and other genes of adaptive nature (Labbate et al., 2009; Moura et al., 2012; Stalder et al., 2012), which increases the possibilities of their selective multiplication.

In other words, exposure to antibiotics, biocides, or heavy metals and a high multiplicity of other different environmental factors results in an increase of cells containing integrons (Gaze et al., 2005, 2011; Wright et al., 2008; Stalder et al., 2012). Moreover, exposure to different antibiotics (aminoglycosides, beta-lactams, fluoroquinolones, trimethoprim, metronidazole) facilitates extensive gene cassette recombination; occasionally involving the SOS-triggered IntI1 integrase over-expression (Guerin et al., 2009; Hocquet et al., 2012). Other recombinational events (often mediated by ISs) influence shuffling of resistance genes contained in different integrons, giving rise to multi-resistance regions (Partridge, 2011) and further facilitating the evolution and selection of the upper-level host vehicles (Domingues et al., 2012). In fact, integrons are not mobile, but are frequently associated with transposons and/or plasmids and therefore should increase in abundance as a result of conjugation or transposition events mediated by Tn*21*-like and IS*26*-like elements. For instance class 1 integrons located in Tn*402*, which are often part of Tn*501*-like transposons on conjugative plasmids, have greatly contributed to the spread of integrons among γ and β-proteobacteria (Tato et al., 2010; Partridge, 2011).

The frequent association of integrons with a variety of specialized DNA recombination systems enhances both transferability and genetic diversification. For instance, insertion sequences (IS) of the IS*110*/IS*492* family as IS*4321* and IS*5075* (members of IS*1111* subgroup) target the terminal inverted repeats of Tn*21* family transposons (Partridge and Hall, 2003; Novais et al., 2010a). The outcome is the initiation of a non-standard transposition resulting in only a single copy appearing in the transposed product (Cain et al., 2010; Martinez et al., 2012). Such mobilization of integrons by specialized transposition is a powerful mechanism for integrons spread in both environmental competent and non-competent bacteria (Domingues et al., 2012; Stokes et al., 2012). Also, IS-mediated mobilization of relevant antibiotic resistance genes contained in the integron contributes to enhance gene expression and mosaic genetic diversity, which should be reflected in higher possibilities of dissemination. That is the case for insertion sequences targeting the pseudo-palindromes of integron *attC* sites, as the IS*1111-attC* group elements of the IS*110*/IS*492* family (Tetu and Holmes, 2008), or IS*4-*like elements as IS*1999 *(Aubert et al., 2006; Post and Hall, 2009; Poirel et al., 2010). Note that the antibiotic-mediated selection of strains, plasmids, or transposons containing integrons necessarily implies selection of IS sequences, and therefore the capture of resistance genes and the combinatorial evolution of resistance platforms. In fact, ISs mobilizing adjacent sequences by rolling-circle (RC) transposition as, IS*Ecp1* and the insertion sequence common regions (IS*CR*s), favor the capture and mobilization of a full series of antibiotic resistance genes leading to complex multi-resistance class 1 integrons (del Pilar Garcillán-Barcia et al., 2001; Garcillán-Barcia and de la Cruz, 2002; Aubert et al., 2006; Toleman et al., 2006; Poirel et al., 2010).

## ANTIBIOTICS AND POPULATIONS OF RESISTANCE TRANSPOSABLE ELEMENTS

Have antibiotics increased the abundance of resistance transposable elements in the microbiosphere? Transposable elements encode an enzyme, transposase, which is required for excising and inserting the mobile element. Transposases (revised in Hickman et al., 2010 and references herein) seem to be the most abundant genes in known sequenced genomes and environmental metagenomes (Aziz et al., 2010).

Among transposases, class II dsDNA transposases constitute the most common group, followed by serine and tyrosine recombinases and RC transposases which are linked to different MGEs (IS, composite transposons, class II transposons, bacteriophages, and genetic islands). The wide spread and maintenance of different classes of transposable elements in bacterial populations has been obviously favored by antibiotic selection because of their association with antibiotic resistance genes. However, most of the contemporary antibiotic resistance transposable elements belong to a few families that have been detected in ancient isolates, often linked to alternative functional roles, thus suggesting antibiotic resistance might have overshadowed previous selection forces.

For instance, some transposable elements, as IS or composite transposons, as Tn*5* or Tn*10*, confer growth rate advantages under different conditions of nutrient availability, enabling populations to rapidly adapt to different physiological conditions (Biel and Hartl, 1983; Hartl et al., 1983; Blot et al., 1994). Also, the highly specialized targeting system of Tn*7* able to selectively direct transposition into both mobile and stationary DNA pools (see later), avoids the occurrence of deleterious insertions and allows the host population or community to recruit genes through a variety of mobile DNAs, thus favoring the adaptation of diverse groups of bacteria to survive or adapt to different conditions (Parks and Peters, 2009; Parks et al., 2009). Selection by different ecological conditions and stressors (including antibiotics) multiplies the chances for expansion, recombination, and diversification (Partridge and Iredell, 2012; Seputiene et al., 2012).

The main group within dsDNA transposases corresponds to DDE transposases (designation given due to the presence of a highly conserved catalytic triad of two aspartate (D) and one glutamate (E) residue), originally identified in the retrovirus integrase and having a role during the transfer of the DNA strand. Most IS families use this catalytic reaction for transposition with the exception of IS*1*/IS*110* and the RCR IS*91*-like elements. IS-DDE transposases have been detected in more than 70 bacterial genera, more than one third being iso-elements (>95% of identity at protein level, >90% at DNA level). They are frequently located on plasmids associated with composite transposons containing antibiotic resistance genes (Merlin et al., 2000).

Tn*7* poses an unique fine-tuned regulated transposition array (TnsABCDE) involved in the regulation of transposition (the core machinery coding for TnsA, TnsB, TnsC) and the mobilization of the element (TnsE, TnsD; Parks and Peters, 2009, Parks et al., 2009). Such regulation allows Tn*7* to use two target-site selection pathways and move to different hosts. Tn*7* belongs to a family of MGE that encodes a transposase and an ATP-utilizing protein (TnsC) that controls the activity of such transposase and often its target site selection. Members of the ATP-subunit superfamily comprise widespread AbR transposons that differ in the transposase (also a DDE enzyme) and the number of proteins in the transposition module (for revision see Craig, 2002). Some examples are Tn*1825*/Tn*1826,* Tn*552*-IS*21*, and Tn*402*–Tn*5053 *(Craig, 2002). All are widely distributed and they are related with trimethoprim and heavy metals resistance (Kholodii et al., 2003; Partridge, 2011). Please note that in this case trimethoprim resistance is the currently recognizable phenotype, but the genes might have been selected before trimethoprim exposure for other reasons (Alonso and Gready, 2006).

Members of the Tn*3* family are mainly derivatives of transposon subfamilies Tn*3* (Tn*3*, Tn*5393*, Tn*5403*) or Tn*501* (Tn*21*, Tn*501*, Tn*1721*) and all of them were already detectable in ancient bacteria from permafrost (Tolmasky, 1990; Graidy, 2002; Kholodii et al., 2003; Mindlin et al., 2008). Members of the Tn*501* subfamily of Tn*3* transposons could have been enriched by mercury exposure, as they carry mercury-detoxifying genes. These genes probably originated in hydrothermal environments, where geochemically derived mercury is at high concentrations (Boyd and Barkay, 2012). Mercury-transposons provide target sites for Tn*501*-type transposons, and include a diversity of Tn*21*, Tn*1696*, and Tn*501* related elements carrying class 1 integrons. Enrichment of Tn*3*-type transposons by environmental pollutants, as heavy metals, might have contributed to increase the connectivity with organisms and genetic platforms harboring resistance genes, eventually included in integrons (most frequently of class 1), and have converted this family in the “flagship” of floating resistance genes (Liebert et al., 1999; Partridge and Hall, 2004; Partridge and Iredell, 2012).

Beside classic antibiotic resistance gene cassettes, emerging beta-lactamase genes as *bla*_VIM_, *bla*_IMP_ *bla*_VEB_, or *bla*_GES_ are located in integrons on different Tn*501* derivatives (Partridge, 2011). Other widespread “new” beta-lactamase genes have been directly recruited by host-specific Tn*3*-like transposons as Tn*1440* carrying *bla*_KPC_ (a Tn*5403*-like transposon from *Klebsiella* which belongs to the Tn*3* subfamily; (Naas et al., 2008).

Such process of gene capture contributes to further increase the number of these transposons, which in turn will facilitate its diversification and eventually novel resistance gene recruitments. Indeed the capture of *bla*_TEM1a_ by Tn*2* led to, in a landscape of high consumption of aminopenicillins in the 60s and 70s of the past century, a huge amplification of this transposon, now widespread in all environments. Other transposons were less-successful, as Tn*1* (with *bla*_TEM-2_), and Tn*3* (carrying *bla*_TEM-1a_; Novais et al., 2010a; Bailey et al., 2011), probably because of the reduced numbers and connectivities (influencing success) of the vehicles in which they were located (plasmids, clones; Cain et al., 2010; Novais et al., 2010a; Partridge, 2011).

Transposable elements also increase in number by inserting extra copies in the host genomes. Of course this might cause an “intergenomic conflict,” as such insertions might affect chromosomal balance and produce mutations, being so, transposons might be a “bitter–sweet” pill for host bacteria (Toleman and Walsh, 2011). This is why genomes have evolved suppressors limiting transposon spread (Pomiankowski, 1999). Eventually, equilibrium is reached by diminishing the transposition frequency. Successful transposons as Tn*3* have this kind of “transposition immunity” to ensure a maximum of two copies per replicon.

On the other hand, transposition might compete with the host genome replication. Some transposases as that of Tn*7* (and possibly Tn*917*) can bind to “processivity factors” involved DNA replication; competition for this interaction could limit their proliferation. However, a benefit for the transposons appear to be derived from the fact that the interaction of transposases with processivity factors favors “target site selection,” so that activation of transposition with Tn*7* (transposon excision and insertion) does not occur until an appropriate target has been identified, most frequently mobile plasmids, providing Tn*7* with a means of spreading to a new host (Parks et al., 2009).

As in the case of integrons, transposable elements are very ancient on Earth, but the very same molecular structures are found in modern resistance-bearing transposons (Bisercić and Ochman, 1993; Mindlin et al., 2005; Vishnivetskaya and Kathariou, 2005; Petrova et al., 2009; Aziz et al., 2010). The acquisition of resistance genes seems to have occurred preferentially by particular transposable elements that were afterward amplified by antibiotics. Interestingly, a number of transposons carrying resistance genes have been recovered from ancient permafrost and seem to have been selected before the antibiotic era (Mindlin et al., 2008). Widespread transposons in our days, as those belonging to Tn*7* or Tn*3* superfamilies, certainly have a very ancient origin. Most probably they were selected by pre-antibiotic forces, increasing their absolute amount, followed by their spread and diversification in different plasmids and organisms. Exposure to early chemotherapeutic agents has reinforced these evolutionary events.

## ANTIBIOTICS AND POPULATIONS OF RESISTANCE MOBILE GENETIC ELEMENTS (PLASMIDS, ICEs)

Have antibiotics increased the abundance of plasmids and ICEs carrying resistance genes in bacterial populations and communities? Conjugative plasmids and ICEs are quite similar genomic objects, in fact they appear to be short-term variants of identical backbone elements (Guglielmini et al., 2011); the main difference is that the replication of ICEs occurs only by integration in the host's chromosome (Wozniak and Waldor, 2010). For instance a close relationship resulting from a common phylogeny can be found between IncA/C plasmids and SXT/R391 ICEs (Wozniak and Waldor, 2010; Toleman and Walsh, 2011). Note that the traditional association of highly transmissible elements with plasmids is not necessarily true.

Plasmids are abundant in nature and consistently isolated from microbial communities of different habitats with and without anthropogenic exposure (Coombs, 2009; Sobecky and Hazen, 2009). In fact, contemporary resistance plasmids are based on plasmid backgrounds existing in the pre-antibiotic era (Datta and Hughes, 1983; Hughes and Datta, 1983). Maintenance of plasmids and ICEs in bacterial populations results from both the selfish features that promote acquisition and persistence within bacterial populations (“the parasitic hypothesis”) and the beneficial effects they confer to individual bacterial hosts and communities (“the evolvability hypothesis”; Werren, 2011). MGEs are increasingly being considered within this multi-hierarchical model, as clonal-, species-, or community-specific mobile elements (Carattoli, 2009; Garcillán-Barcia et al., 2009; Lim et al., 2010; Shearer et al., 2011; Heuer et al., 2012; Williams et al., 2012; Clewell et al., 2013; Guglielmini et al., 2013). Between and also within these hierarchical levels, plasmids may eventually evolve toward mutualism (Rankin et al., 2011b). Plasmids might assure their permanent linkage with a particular host, bacterial lineage, or multi-specific community by post-segregation killer strategies that cause the death of non-carrying bacterial offspring. This is caused by toxin–antitoxin (TA), restriction-modification (R-M), or clustered regularly interspaced short palindromic repeats (CRISPR) systems, and also probably by “plasmid domestication,” which is produced by changes either in the plasmid and host genome that lead to a more stable coexistence (Bouma and Lenski, 1988; Jones, 2010; Marraffini and Sontheimer, 2010; García-Quintanilla and Casadesús, 2011).

The diversity of bacterial communities, the relative population densities of their components, the spatial separation, and nutrient availability greatly influence plasmid host-range, content, and transferability (Coombs, 2009). It is interesting to suggest that the selective processes exerted by antibiotics will modify bacterial diversity and population densities, forcing the coexistence of plasmids and particular hosts, favoring recombination and other processes that lead to plasmid domestication (Boyd et al., 1996). The robustness of interactions between particular plasmids and particular clones is shaped by epistatic processes (Silva et al., 2011; San Millán et al., 2012) mediated by nucleoid-associated transcriptional regulators (Doyle et al., 2007; Yun et al., 2010; Fernández-Alarcón et al., 2011; Humphrey et al., 2012) and the clonal interferences that might result from these interactions (Hughes et al., 2012). A robust interaction is reflected in a non-cost or even negative-cost coexistence, and will tie the fate of plasmid density to the abundance of their specific bacterial hosts, resulting in a necessary “in-host” plasmid evolution linked to “with plasmid” host evolution (Dionisio et al., 2005; Halary et al., 2010).

A major topic in plasmid population biology is the consideration of advantages and inconveniences of plasmid or ICE broad-host-range. It is of note that host-range does not necessarily mean transfer ability to a particular host or long-term maintenance in bacterial populations, but stable replication in a new host (De Gelder et al., 2007; Suzuki et al., 2010). Certainly, broad-host-range conjugative plasmids favor the penetration of adaptive traits as “new” antibiotic resistance genes (Fernández-Alarcón et al., 2011; Sen et al., 2011; Hamprecht et al., 2012; Heuer and Smalla, 2012) and, in turn, antibiotics can favor the abundance of these plasmids promoting transfer and by selection of plasmid containing bacteria (Lang et al., 2012). The frequent observation of different systems of replication (recognizing host primases) in the same plasmid suggests adaptive ways of increasing host-range (Del Solar et al., 1996; Clewell et al., 2013). Globally spread plasmids identified in hospitals, soils, agriculture, and marine habitats have a complex mosaic structure that reflects inter-genomic historical adaptations to phylogenetically distant bacterial hosts (Schlüter et al., 2007; Norberg et al., 2011; Heuer et al., 2012; Partridge and Iredell, 2012). In addition, broad-host-range plasmids lacking transfer systems can be transferred to phylogenetically close or distantly related bacteria by helper conjugation systems located in narrow-host-range plasmids containing a conjugation system (Smorawinska et al., 2012).

Long-term maintenance and dispersion of broad-host-range plasmids in bacterial populations and communities seems to be related with the local availability of hosts (De Gelder et al., 2007), but also with the “social interactions between plasmids” eventually leading to unbearable costs for their hosts (Smith, 2012). Eventually, exclusion mechanisms between plasmids (one plasmid excludes the incoming one) might be softened by inter-plasmid recombination that might result in hybrids able to evade exclusion. CRISPR is a genetic interference system by which bacteria with CRISPR regions carrying DNA copies of previously encountered plasmids can abort the replication of plasmids with these sequences. Hypothetically that might favor plasmid dispersal among different strains, providing a weak selective advantage for the host cell (Levin, 2010), although an increased benefit could be predicted for host coalitions, as genetic exchange communities (GECs; see later). This system also controls phages, but the possible populational interactions (competition–cooperation) between phages and plasmids have scarcely been investigated.

Antibiotic exposure might have increased the absolute number of plasmids with resistance determinants in bacterial populations due to the selection of clones harboring them. The possibility that broad-host conjugative plasmids have been submitted to a more effective enrichment than narrow-host plasmids (because of selection in multiple hosts and environmental compartments) poses an interesting question. Eventually, the biological cost of resistance plasmids for the host could be compensated by higher transmission (Garcillán-Barcia et al., 2011). As Stokes and Gillings pointed out, an increase in the general *tempo *of resistance genes dissemination is highly probable, due to selection of cells with inherently higher rates of lateral transfer (Stokes and Gillings, 2011).

Finally, we can consider bacteriophages as mobile mediators of inter-bacterial transfer of resistance genes. Also in this case antibiotics might modulate phage–bacteria population dynamics by processes as “phage-antibiotic synergy,” a non-SOS mechanism of virulent phage induction caused by exposure to sub-inhibitory concentrations of beta-lactams (Comeau et al., 2007; Allen et al., 2011; Looft et al., 2012). Antibiotics promote the number of phages and pro-phages linked to antibiotic resistance platforms, favoring dissemination of these platforms, and consequently amplifying the dissemination of resistance and virulence genes (Allen et al., 2011).

## ANTIBIOTICS AND POPULATIONS OF BACTERIAL CLONES AND SPECIES

Bacterial clones are constituted by isolates that have a close common phylogenetic origin. High-risk clones are defined as clones with an enhanced ability to colonize, spread, and persist in a variety of niches, which acquire adaptive traits that increase pathogenicity or antibiotic resistance (Baquero and Coque, 2011). They constitute the main vehicles dispersing antibiotic resistance at a global scale (Willems et al., 2011; Woodford et al., 2011). Examples of these high-risk clones are *E. coli* ST131 (phylogroup B2), ST155 and ST393 (phylogroup B1), or ST69, ST405, and ST648 (phylogroup D); *K. pneumoniae* ST258, ST14 or ST37; *Enterococcus faecium*, ST18, ST17, ST78; *Enterococcus faecalis *ST6; *S. aureus*, ST45, ST5, ST8, ST30, or ST22. A number of these clones were ancient lineages, well-adapted to colonization and transmission between particular hosts, that acquired antibiotic resistance and consequently enhanced capabilities of dispersal (McBride et al., 2007; Brisse et al., 2009; Chambers and Deleo, 2009; Willems et al., 2012). Multiplication and spread of highly successful clones implies multiplication and spread of all the antibiotic resistance genes and platforms they contain, increasing their absolute numbers. In fact, it is not unusual that a single successful clone might contribute to the spread of different plasmids, genetic platforms, and resistance genes, both in Gram-positives (Chambers and Deleo, 2009) and in Gram-negatives (Carattoli, 2009; Andrade et al., 2011; Woodford et al., 2011; Novais et al., 2012a; Partridge and Iredell, 2012). Such multi-lateral collaboration probably contributes to the local ecological success of variants arising in these clones, leading to a clonal diversification (clonalization) which assures a long-term permanence in complex adaptive landscapes, following the “never put all the eggs in the same basket” principle (Wiedenbeck and Cohan, 2011; de Regt et al., 2012; Freitas et al., 2013). Focusing only on mutational evolution, it has been suggested that there is an acceleration of emergence of bacterial antibiotic resistance in connected microenvironments (Zhang et al., 2011; Hallatschek, 2012) but this might also occur in the case of gene flow.

Local clonalization might result in a restricted gene flow among resulting subpopulations (Willems et al., 2012). However, recombinational events might spare those regions required for adaptation to local microniches, assuring divergence, and be maintained for other regions (ecological speciation with-gene-flow; Via, 2012). The increased recovery of isolates belonging to high-risk clonal complexes of important human pathogens as *E. coli, S. aureus, E. faecalis*, or *E. faecium,* that cause both human infections and mucosal colonization, and even the expansion of these clones to novel hosts is most probably related with the acquisition of antibiotic resistance genes (Hidron et al., 2008; Baquero, 2012; Novais et al., 2012a, 2013)

An interesting question is if the selection of particular clones because of antibiotic resistance might decrease the overall clonal diversity as might be inferred from recent studies (Ghosh et al., 2011). Actually, that might be compensated by clonal diversification, in a “*ex pluribus unum/ex unibus plurum*” evolutionary dynamics (Baquero, 2011). It is easy to conclude that any outbreak produced by antibiotic resistant bacteria will locally enrich the involved evolutionary units, facilitating further events of antibiotic resistance development and possibly transmission (Chambers and Deleo, 2009; Freitas et al., 2013).

## ANTIBIOTICS AND POPULATIONS OF BACTERIAL COMMUNITIES

Imbedded into the high complexity of the microbiomes, of humans and animals, in the soil or in the water sediments, it is possible to recognize “clubs” of bacterial clones and species where genes and genetic platforms circulate via lateral transfer, the GECs. In a very restrictive manner, Skippington and Ragan (2011) have recently defined a GEC as a group of organisms (entities) in which each entity has over time both donated genetic material to, and received genetic material from every other entity in that GEC, via a path of lateral gene transfer. What do the members of such clubs have in common? Network modeling and co-occurrence statistical approaches indicate that lifestyle and shared environments, functional complementarities, and most probably, continued physical clustering (granularity) determine the size and connectivity of GECs (Freilich et al., 2010; Smillie et al., 2011; Faust and Raes, 2012; Faust et al., 2012). In many cases, this also means a closed shared phylogeny (Skippington and Ragan, 2012). In fact, GECs members are linked not only by genetic exchanges, but also by metabolic and functional cooperation, providing a certain ecological compartmentalization inside particular microbial megasystems (as intestinal microbiota; Faust and Raes, 2012; Faust et al., 2012). We can consider here some kind of cooperative “niche construction” (Laland et al., 1999; Kylafis and Loreau, 2011). Genetic transfer, particularly considering mixed-granular “surface-associated populations” with a kind of “lattice reciprocity” (Zhong et al., 2010, 2012) might assure a “collective” adaptation of such functional GEC units, increasing relatedness among members and fixing common evolutionary boundaries (Nogueira et al., 2009; Rankin et al., 2011a,b). The development of more studies on the “physics” of genetic transfer, is certainly advisable, for instance, to investigate to what extent genetic transfer can be influenced by mixing movements or the viscosity or fluidity of the surrounding medium, as in the intestinal content (particularly during enteric diseases) or in water bodies, influencing cell-to-cell contacts and therefore GECs integrities (Zhong et al., 2010; Jeffery et al., 2012).

An important topic is the possibility of “multiclonal GECs” as a form of organization of the lifestyle of a particular species or closely phylogenetically related coalitions in changing environments (Skippington and Ragan, 2012), where different sub-specific ecotypes exploiting neighbor nano-niches (Wiedenbeck and Cohan, 2011) and taking advantage of a common “public good” are frequently encoded by conjugative elements (Norman et al., 2009; Rankin et al., 2011b). The distribution among GECs members of such plasmid-mediated “public goods” is favored by common characteristics in the consortium, as nearly identical immune phenotypes mediated by CRISPR, or common DNA uptake mechanisms or quorum sensing responses. Plasmid-mediated common benefits will probably lead to GEC-plasmid coevolution (Skippington and Ragan, 2012). Along the same line, addiction-type TA complexes can spread on plasmids, favoring coexistence and/or competition in spatially structured environments (Rankin et al., 2012) highlighting the role of kin effects in GECs selection (taking “kin” in a wider sense than just intra-specific ties). It is of note, however, that possibly most organisms and environments might act as conduits for resistance gene flow (Stokes and Gillings, 2011), acting as “sources” from where resistance genes are directed to GECs, acting as “sinks,” according to the Perron et al. (2007) metaphor. The possibility of “go-between” organisms traveling from GECs of different microbiotic systems (humans, animals, rhizosphere, and water sediments) should be taken into consideration, as they might contribute to the inter-environmental globalization of antibiotic resistance genes. The existence of “ubiquitous” organisms or species able to transit in different environments has been suggested (Fondi and Fani, 2010; Freilich et al., 2010; Tamames et al., 2010). Candidates for efficient “go-between” organisms are groups of the same bacterial species but specialized in particular environments, as the case of human or animal-adapted versus environmental *E. coli* clades, where probably only ecological barriers prevent gene flow (Freilich et al., 2010; Luo et al., 2011). Mixing of human or animal derived water effluents into the environment, a practice that is surprisingly perpetuated even in modern societies will facilitate conduits for resistance gene flow (Baquero et al., 2008; Czekalski et al., 2012; Lupo et al., 2012). Such flow occurs because of the confluence of human microbiota from different human hosts, different animals and the indigenous environmental microbiota.

Among these GECs, the most relevant for the transmission of antibiotic resistance are those including species from Gammaproteobacteria (as *E. coli*) and Firmicutes (as *Enterococcus*; Antonopoulos et al., 2009; Freilich et al., 2010; Skippington and Ragan, 2011; Faust et al., 2012). These groups of organisms are enriched in the microbiota during antibiotic exposure (Antonopoulos et al., 2009; Sommer et al., 2009). Antibiotic-mediated reduction in number or loss of some species, favors bacterial species able to explore and temporarily exploit empty niches due to short generation times (Allen et al., 2011; Looft and Allen, 2012). Antibiotic exposure will increase the absolute number (overgrowth) of GECs-associated organisms, for instance, by antibiotic exposure of the infant gut (Fouhy et al., 2012; Looft and Allen, 2012). Probably the same might occur in environmental GECs submitted either to antibiotic pollution or sanitation procedures (Baquero et al., 2008). For instance, metagenomic analysis indicates that drinking water chlorination could concentrate populations containing insertion sequences, integrons, and antibiotic resistance plasmids (Shi et al., 2013). These effects will contribute to the dissemination of resistance genes and the genetic platforms in which they are located.

The identification of GECs among members of the “microbial guilds” in the three identified major microbiome “enterotypes” (Arumugam et al., 2011) needs to be investigated. These major enterotypes (clusters of species) should probably be tightly maintained into host populations, and therefore the local spread of antibiotic resistance genes could serve to maintain their integrity in an antibiotic-polluted environment. At the same time, low-abundance enterotype-species, but providing critical functions, could have low possibilities of getting resistance genes in the absence of GECs. Consequently, a deep and permanent contamination by antibiotic resistance genes of the normal microbiota of humans and animals is a reasonable possibility. Complex microbiota of humans and animals are reproducible systems, not only along time in the same individual, but also across individuals. These systems are frequently based in a microbiotic “core” composed by organisms belonging to a relatively small number of phylogroups, and probably metabolically linked with the host (Dethlefsen et al., 2006; Ley et al., 2006; Marchesi, 2011). Newborns have a sterile gut, but the human microbiota is “reproduced” with a relatively low number of variations on each of them (Baquero and Nombela, 2012; Vallès et al., 2012). Possibly there is also an “epidemiology of bacterial consortia” even in hospitals, which remains to be investigated. Exposure to antimicrobial agents might affect the frequency and absolute number of GECs; in a number of cases, antibiotic resistance might contribute to the temporal stability and resilience of microbiomes in an antibiotic-polluted environment (Allison and Martiny, 2008; Antonopoulos et al., 2009). Of course that occurs at the expenses of maintenance and spread of the full range of antibiotic resistance evolutionary units.

Finally, we cannot discard individual variations in the microbiotic communities caused by diet, host genetics, particular illnesses, inflammation, or infectious agents including viruses and parasites which might lead to microbial communities more prone to capture and propagate antibiotic resistance (Marchesi, 2010; Claesson et al., 2012; Looft and Allen, 2012). For instance, a high-fat diet determines the composition of the murine gut microbiome independently of obesity, with an increase of Proteobacteria and Firmicutes, heavily involved in resistance gene mobilization (Hildebrandt et al., 2009; Tagliabue and Elli, 2012). In several microbiota communities studied in the elderly, the proportion of phylum Proteobacteria, very active in the mobilization of antibiotic resistance genes and vehicles, was ten times higher than average (20 versus 2%; Claesson et al., 2011).* E. coli* numbers are higher in the microbiota of women with excessive weight gain than in women with normal weight gain during pregnancy (Santacruz et al., 2010).

A number of surgical interventions (as surgery for morbid obesity) increases Proteobacteria even in a higher proportion (50 times increase; Li et al., 2011; Graessler et al., 2012). Unfortunately, these populational microbiotic shifts favoring the active populations and communities contributing to the emergence, dispersal and maintenance of antibiotic resistance might also occur as a consequence of undernutrition (10 times increase in Proteobacteria, 46 versus 5% in healthy children in Bangladesh; Monira et al., 2011). Possibly the deleterious effect of antibiotics in promoting antibiotic resistance will be significantly increased. Finally, it could be considered, under certain circumstances, as during the colonization of the neonatal intestinal tract, that rapidly growing populations might be more prone to the dissemination of antibiotic resistance. Also the unexpected possibility of resistance gene exchange between Actinobacteria (*Bifidobacterium* belongs to this group!) and Gammaproteobacteria has been recently shown under the same conditions (Tamminen et al., 2012).

## ANTIBIOTICS IN THE ANTHROPOCENE: EFFECTS ON GLOBAL ECOLOGY AND EVOLUTION

Evolution is a natural trend of complex systems, and might be accelerated by changing and stressful conditions. The Anthropocene is the current human-dominated geological epoch where nature is changed and stressed by the action of humans (Zalasiewicz et al., 2011; Biermann et al., 2012). Industrial antibiotics are a paradigmatic example of substances exerting a powerful effect of anthropogenic origin on the bacterial communities of the microbiosphere. Not only most of these substances are unspecifically killing bacterial organisms, and selecting for resistance, but directly influence the mechanisms of genetic variation (mutation, recombination, transposition, modularization, gene transfer; Baquero, 2009; Gillings and Stokes, 2012).

Such effects on microorganisms will be further enhanced by a diversity of other anthropogenic effects as the release of biopharmaceuticals, biocides, heavy metals, industrial and agricultural residues, and plastic materials or changes in the environmental conditions. The mixing of bacterial populations (human organisms with other human, animal, or environmental organisms), that makes the emergence and spread of resistance possible is also favored by poor sanitation, facilitating contact of human or animal sewage with the soil. Some of these effects might escalate with other anthropogenic effects as destruction of diversity in food animals (Baquero, 2012) or even global warming (Baquero et al., 2008; Baquero, 2009; Balbus et al., 2013). The fight against antibiotic resistance should focus not only on acting on its appropriate usage in human and veterinary medicine, but by considering possible initiatives at ecological and evolutionary levels, as eco-evo drugs and strategies (Baquero et al., 2011) in accordance with the environmental distribution of bacterial organisms (Tamames et al., 2010), in the scope of progressing toward a protective and restorative planet medicine (Baquero, 2009).

## Conflict of Interest Statement

The authors declare that the research was conducted in the absence of any commercial or financial relationships that could be construed as a potential conflict of interest.
